# Academic performance of students who underwent psychiatric treatment at the students’ mental health service of a Brazilian university: Desempenho acadêmico de alunos que se submeteram a tratamento psiquiátrico no serviço de saúde mental para estudantes de uma universidade brasileira

**DOI:** 10.1590/1516-3180.2016.017210092016

**Published:** 2017-01-05

**Authors:** Cláudia Ribeiro Franulovic Campos, Maria Lilian Coelho Oliveira, Tânia Maron Vichi Freire de Mello, Clarissa de Rosalmeida Dantas

**Affiliations:** I MD. Psychiatrist, Master’s Degree Student, Students’ Psychiatric and Psychological Service, Faculdade de Ciências Médicas da Universidade Estadual de Campinas (FCM-Unicamp), Campinas (SP), Brazil.; II MSc. Psychologist, Students’ Psychiatric and Psychological Service, Faculdade de Ciências Médicas da Universidade Estadual de Campinas (FCM-Unicamp), Campinas (SP), Brazil.; III MD, PhD. Psychiatrist, Students’ Psychiatric and Psychological Service, Faculdade de Ciências Médicas da Universidade Estadual de Campinas (FCM-Unicamp), Campinas (SP), Brazil.; IV MD, PhD. Psychiatrist and Professor, Department of Medical Psychology and Psychiatry, Faculdade de Ciências Médicas da Universidade Estadual de Campinas (FCM-Unicamp), Campinas (SP), Brazil.

**Keywords:** Mental disorder, Counseling, Universities, Students, Mental health

## Abstract

**CONTEXT AND OBJECTIVE:**

University students are generally at the typical age of onset of mental disorders that may affect their academic performance. We aimed to characterize the university students attended by psychiatrists at the students’ mental health service (SAPPE) and to compare their academic performance with that of non-patient students.

**DESIGN AND SETTING:**

Cross-sectional study based on review of medical files and survey of academic data at a Brazilian public university.

**METHODS:**

Files of 1,237 students attended by psychiatrists at SAPPE from 2004 to 2011 were reviewed. Their academic performance coefficient (APC) and status as of July 2015 were compared to those of a control group of 2,579 non-patient students matched by gender, course and year of enrolment.

**RESULTS:**

37% of the patients had had psychiatric treatment and 4.5% had made suicide attempts before being attended at SAPPE. Depression (39.1%) and anxiety disorders/phobias (33.2%) were the most frequent diagnoses. Severe mental disorders such as psychotic disorders (3.7%) and bipolar disorder (1.9%) were less frequent. Compared with non-patients, the mean APC among the undergraduate patients was slightly lower (0.63; standard deviation, SD: 0.26; versus 0.64; SD: 0.28; P = 0.025), but their course completion rates were higher and course abandonment rates were lower. Regarding postgraduate students, patients and non-patients had similar completion rates, but patients had greater incidence of discharge for poor performance and lower dropout rates.

**CONCLUSION:**

Despite the inclusion of socially vulnerable people with severe mental disorders, the group of patients had similar academic performance, and in some aspects better, than, that of non-patients.

## RESUMO

**CONTEXTO E OBJETIVO:**

Estudantes universitários geralmente estão na faixa etária típica do início de transtornos mentais que podem afetar seu desempenho acadêmico. Tivemos como objetivos caracterizar os estudantes atendidos por psiquiatras em serviço universitário de saúde mental para alunos (SAPPE) e comparar seu desempenho acadêmico com o de alunos não pacientes.

**DESENHO E LOCAL:**

Estudo transversal baseado em revisão de prontuários e levantamento de dados acadêmicos em uma universidade pública brasileira.

**MÉTODOS:**

Prontuários de 1.237 estudantes assistidos por psiquiatras do SAPPE entre 2004 e 2011 foram revisados. Seu coeficiente de rendimento (CR) e status acadêmicos em julho de 2015 foram levantados e comparados aos de um grupo de controle com 2.579 alunos não pacientes, pareados por sexo, curso e ano de matrícula.

**RESULTADOS:**

37% dos pacientes tiveram acompanhamento psiquiátrico e 4,5% fizeram tentativas de suicídio prévios ao atendimento pelo serviço. Os diagnósticos mais frequentes foram depressão (39,1%) e transtornos fóbico-ansiosos (33,2%). Transtornos mentais graves, como o psicótico (3,7%) e o bipolar (1,9%), foram menos frequentes. Entre os pacientes dos cursos de graduação, o CR médio foi levemente inferior (0,63; desvio padrão, DP: 0,26; *versus* 0,64; DP: 0,28; P = 0,025) que o de não pacientes, mas suas taxas de conclusão do curso foram maiores e as de evasão, menores. Na pós-graduação, as taxas de conclusão foram semelhantes, mas pacientes tiveram maior frequência de desligamento por baixo desempenho acadêmico e menor de desistência.

**CONCLUSÃO:**

Mesmo incluindo pessoas socialmente vulneráveis e com transtornos mentais graves, o grupo de pacientes teve desempenho acadêmico semelhante e, em alguns aspectos melhor, do que o de não pacientes.

## INTRODUCTION

Admission into university is indicative of certain capabilities among young adults, which allowed them to complete high school and pass the entrance examinations. Those conditions are not limited to cognitive traits, but also include access to information and an evolved state of internal and external mental organization and structures. On the other hand, this phase of life brings new challenges such as living away from family, making new friends and adapting to a new level of academic requirements. It is also an age at which outbreaks of various mental disorders frequently occur.^1^^,^^2^^,^^3^ For such reasons, since the beginning of the twentieth century, a number of universities in the United States and Europe have created internal services for student mental health care.^4^ Such concerns have also motivated several institutions in Brazil to establish their own services for the same purpose.^4^

Campinas State University (Universidade Estadual de Campinas, Unicamp) is a Brazilian public university, founded in 1966.^5^ In 2011, the university had 27,783 regular students, of whom 60.04% were undergraduates.^7^ The university’s Psychological and Psychiatric Service for Students (Serviço de Assistência Psicológica e Psiquiátrica ao Estudante, SAPPE), which is the mental health service on the campus, was created in 1987. The service is structured such that psychiatrists provide medical support for psychological treatment, thus attending to the most severe cases. During the survey period of the present study, psychiatric consultations accounted for around 15% of all attendance provided by the service.^6^^,^^7^

A study conducted within SAPPE^8^ reviewed the medical files of all students who sought the service between 1987 and 2003. It found that students who were dependent on scholarships and those living in student housing belonging to the university were overrepresented in relation to the total number of university students. This indicated that mental care on the campus was more important to students whose economic conditions were unfavorable. Another study conducted in 2011^9^ surveyed the group of students who sought the service for a second time after completing the initial treatment. It identified unfavorable economic situation, academic difficulties, early seeking of the service for first attendance and low self-esteem as the main factors associated with returning to the service.

In 2005,^6^ the university debuted its Affirmative Action and Social Inclusion Program (Programa de Ação Afirmativa e Inclusão Social, PAAIS), a series of measures following federal government guidelines for expansion of social inclusion programs. Initially, it was intended to cover 30% of new undergraduate students, but was recently expanded to 50% of entrants.^10^ In the light of previous studies, it is reasonable to expect that the resulting growth of the vulnerable university population will imply an expansion of the number of students who are dependent on healthcare services provided by the university.

## OBJECTIVE

Our aim was to characterize the patients treated by psychiatrists at SAPPE, describing some of their socioeconomic and clinical attributes, and to compare some of their academic performance indicators with those of their colleagues who were not assisted by the service. Our purpose was to move a few steps further forward in gathering inputs for planning, not only of the mental health services themselves, but also of broader strategies that may be needed to address the ongoing changes affecting the student population.

## METHODS

The National Commission for Research Ethics (Comissão Nacional de Ética em Pesquisa, CONEP) approved this descriptive, retrospective study based on medical file review. We reviewed the medical files of all undergraduate and postgraduate students attended by the mental health service of the campus between January 2004 and December 2011 and identified 1,237 cases in which a student underwent psychiatric consultation, comprising 769 undergraduate and 468 postgraduate students.

Through examination of the records, we obtained the following: sociodemographic information consisting of gender, age, marital status, origin and type of income; prior clinical information consisting of prior psychiatric care, assistance by hospital psychiatric services and suicide attempts; and information gathered during treatment, comprising the ascribed diagnosis and prescribed medications, assistance or hospitalization by the hospital psychiatric service and suicide attempts. The data collection was carried out between August 2014 and February 2015.

The university’s academic board (Diretoria Acadêmica, DAC) provided the academic data. The information referred to the first half of 2015 and consisted of the academic status and the academic performance coefficient (APC). The APC is an index used by the university to measure students’ overall academic performance along the course, calculated from the grades obtained and the number of credits in each subject of the course. It is similar to the grad-point average (GPA), except that it is scaled from -1.0000 to 1.0000 for undergraduate courses and from 0.0000 to 4.0000 for postgraduate courses. The APC is best suited for evaluating the performance of undergraduate students, given the heterogeneity of master’s and doctorate programs in terms of structure and evaluation methods. Therefore, we assessed those two education levels (undergraduate and postgraduate) separately and for postgraduate students, we analyzed only academic status.

With regard to establishing parameters for evaluating academic indicators, we asked DAC to set up a control group through random selection of at least two other students from the same course and from the same semester of enrollment for each patient of the service. They were also asked to preserve the same gender proportion found in the group of assisted students.

Specifically for the undergraduate courses, we compared 769 assisted students with a control group of 1,514 students who did not receive assistance from SAPPE, and then, separately, 468 assisted postgraduate students with a control group of another 1,065 postgraduate students who did not attend this service. Within the control group, the proportions of students affected by mental diseases and of students already subject to mental health care outside of SAPPE are unknown to us. The reason for selecting a comparison group with twice the number of students was to mitigate the distortions that might have arisen from that factor. We performed the comparisons separately according to course level (undergraduates and postgraduates), using the chi-square test for categorical variables and the Mann-Whitney test for comparisons of APC. The latter was calculated only for undergraduates.^11^^,^^12^ The level of significance was 5%.

## RESULTS

### Sociodemographic data

The average age of the students when they were first assisted by psychiatrists at SAPPE was 25.3 years, with standard deviation (SD) of 5.8, median of 24.0, minimum of 17 and maximum of 60 years. They were mostly women (56.9%), singles (81.8%), from the state of São Paulo (71.8%) and living in dwellings shared with other students (*repúblicas*) (35.3%). A scholarship was the main source of income for 41.1% of the students, while 31.5% lived supported by family resources and 18.8% from their own savings. Only 20.8% of these students attended night classes.

Table 1 shows some of the sociodemographic attributes, as well as some general academic data such as the fields of study and the distribution between undergraduates and postgraduates.

**Table 1:**
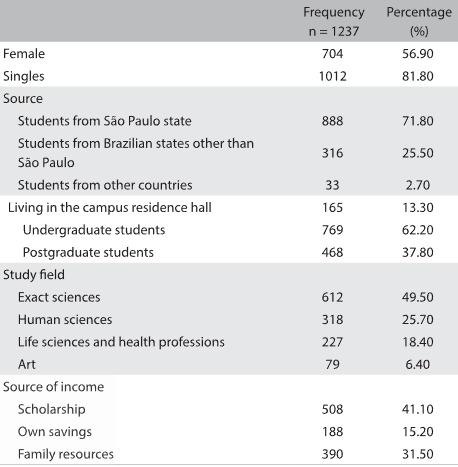
Characteristics of mental health campus service clients

### Clinical data

When the students sought psychiatric care at SAPPE for the first time, 37.0% (n = 454) of them had undergone some prior psychiatric treatment and 2.8% (n = 34) had already gone through hospital psychiatric services. The data showed that 4.53% (n = 56) of the students had made suicide attempts before seeking the service. Among these, 19 (1.5%) had made more than one attempt. During the period of treatment at SAPPE, 1.78% (n = 22) attempted suicide. Six students made more than one attempt. The majority of the students assisted by psychiatrists (74.6%; n = 923) were also under simultaneous psychotherapeutic care (Table 2).

**Table 2:**
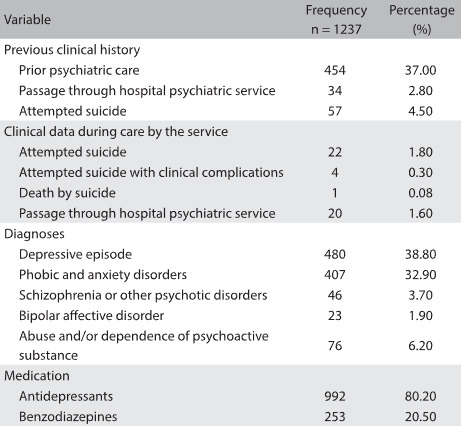
Clinical attributes

The most frequent diagnoses were depressive episodes (n = 480; 38.8%); anxiety and phobic disorders (n = 407; 32.9%); abuse of and/or dependence on psychoactive substances (n = 76; 6.2%); schizophrenia and other psychotic disorders (n = 46; 3.7%); and affective bipolar disorder (n = 23; 1.9%).

The drugs most prescribed were antidepressants, prescribed to 80.2% of the patients (n = 992), followed by benzodiazepines, prescribed to 20.5% of the patients (n = 253). The average number of psychiatric consultations per student was 8.3 (SD = 9.5; median = 5).

### Academic performance of the assisted students compared with that of those who did not attend the service

The undergraduate students who received psychiatric care at SAPPE had slightly lower mean academic performance coefficient (APC) when compared to the control group of non-patient students (0.63, SD = 0.26, versus 0.64, SD = 0.28). Although small, this difference was statistically significant (P = 0.025, Mann-Whitney test).

By the end of the first half of 2015, among the group of undergraduate patients, 515 students (67.0%) had completed their courses, 128 (16.7%) had abandoned the course; 82 (10.7%) had been discharged because of low academic performance, and 42 students (5.5%) had courses still in progress. In the undergraduate control-group, the rate of course completion was significantly lower (57.9%), and the rate of course abandonment higher (27.8%), chi-square test, P < 0.0001 (data presented in Table 3).

**Table 3:**
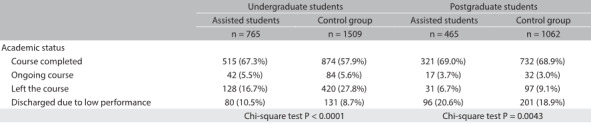
Academic status by the end of the first half of 2015

Among postgraduate students assisted over the surveyed period, only 17 students (3.7%) were still enrolled in their courses at the end of the first half of 2015. Just over two-thirds (69.0%; n = 321) had completed the course, while 6.7% (n = 31) had left the course before its conclusion. Among the latter, seven students did so because they transferred to another course. This number also accounts for direct progression from a master’s to a doctoral course. Around one in five of the assisted postgraduate students (20.6%; n = 96) was clearly discharged for poor academic performance. Three were cut off from their programs but rejoined later on, solely to defend their dissertation or thesis, as allowed by Unicamp’s master’s and doctoral program statutes. In the postgraduate control group, the course completion rate was very similar (68.9%); the rate of drop-out was a little higher (9.13%) and the rate of discharge due to poor academic performance, a little lower, but no significant difference was found (chi-square test, P = 0.3538) (Table 3).

## DISCUSSION

To the best of our knowledge, this is the first study conducted in Brazil to address the academic performance of university students who underwent psychiatric treatment. This study also examined the association between psychiatric disorders in general and academic performance.

With regard to gender distribution at the university during the period 2004-2011,^6^ men formed the majority (55%) of the total student population of the university. This indicates that there was female overrepresentation among the clientele served by SAPPE. This finding is consistent with other studies that correlated demand for mental health care and gender.^8^

Admittance to campus halls of residence follows socioeconomic selection criteria and extends to only about 3.0% of the university students.^6^ About 13% of the assisted students were resident there: a clear overrepresentation that is corroborated by previous studies carried out in this service.^8^ The same applies to the overrepresentation of students whose main source of income was scholarships.

The inverse relationship between mental disorders and economic standard of living is one of the most consistent results from epidemiological population studies and studies on primary care, not only in Brazil but also internationally. However, the relationship between mental health/illness and social vulnerability is very complex and requires deep reflection and contextualization in order to be understood. A simplistic form of logic that correlates “madness” and “poverty”, thereby reinforcing stigma and prejudice with regard to the least favored population, is a pitfall to be avoided.^13^

The fact that a considerable number of students underwent psychiatric care before seeking the service for the first time is open to several interpretations. It could be an indication of greater severity, but could also be a consequence of reduction of stigma, which would stimulate an earlier search for care and might possibly have contributed towards success in being admitted into the university.

The most frequent diagnosis was depressive episodes, followed by anxious and phobic disorders. Among the studies conducted in Brazil, we did not find any centered on students who underwent psychiatric treatment that allowed us to establish direct comparisons regarding the prevalence of different mental disorders. The majority of the studies to which we had access screened either the general population or some specific group (mostly healthcare-related courses) for the prevalence of mental disorders. Some other published papers have pointed out that only a relatively small proportion of the students affected by mental disorders seek and receive clinical attention.^14^^,^^15^^,^^16^^,^^17^ This applies especially to substance-related disorders^18^ and might explain the somewhat low prevalence of those disorders in our sample. Also at lower proportions, we found students with diagnoses commonly regarded as severe mental disorders, such as schizophrenia and other psychotic disorders, along with bipolar disorder. The prescription records relating to different classes of psychopharmacological drugs showed a good proportional relationship to the distribution of diagnoses found.

The frequencies of suicide attempts and instances of care provided by hospital psychiatric services during the course of psychiatric treatment at SAPPE were lower than those reported previously to the treatment at the service (1.78% and 1.5% versus 4.53% and 2.8%, respectively). Whether this decrease might be attributable to a potential protective effect from the psychiatric and psychotherapeutic care received is a question that we cannot positively answer without further research.

The comparisons of academic parameters show that the assisted undergraduate students had an academic performance coefficient (APC) that was only slightly below their colleagues in the control group. Taking into account both the negative impact of mental illness on academic performance^19^ and the fact that there was an overrepresentation of students in economically and socially vulnerable situations among the clients at SAPPE, we consider that this result is a very positive outcome. We might interpret it as suggestive of the effectiveness of providing mental health care on the university campus. Nevertheless, caution is required given that we are unable to make any assertions regarding the proportion of students in the control group who might have been affected by mental illness without receiving treatment either within or outside of our service.

We were positively surprised by the fact the assisted undergraduate students presented a higher course completion ratio than the control group. A study in 2010^19^ evaluated the independent associations between psychiatric disorders among college freshman and the failure to complete the college course. Five diagnoses were positively and significantly associated with failure to graduate: bipolar I disorder, marijuana use disorder, amphetamine use disorder, cocaine use disorder and antisocial personality disorder. The authors suggested that the benefits of prevention, detection and treatment of psychiatric illness might therefore include higher college graduation rates. The fact that the students attended by psychiatrists at SAPPE performed well, concerning dropout rates in comparison with their colleagues in the control group, might also be considered to be a good outcome, given that graduation from a university course can generally be considered to be an important achievement. It needs to be borne in mind, however, that at an individual level, academic dropout is not necessarily a bad outcome. For instance, although abandoning a course may seem to be an unfavorable event, if the student does this because he has the opportunity to enter another institution that is more aligned with his aspirations, this will indeed be a favorable outcome.

The course completion rate among the assisted postgraduate students was almost equal to that of the control group. Postgraduate courses have more stringent deadlines than undergraduate courses, ranging from 12 to 30 months for a master’s degree and 24 to 48 months for a doctorate. Delayed completion of the course results in automatic discharge from the program. The dropout rate among the assisted postgraduate students was similar to that of their colleagues in the control group.

Our study design did not allow us to attribute the positive outcomes that we found solely or directly to the care provided by SAPPE or to any other known factor. Nonetheless, we consider that our results are encouraging with regard to the continuity of efforts towards providing mental health care and other forms of social assistance to university students.

All the limitations of the methods of retrospective medical record reviews need to be taken into consideration in this study. There is no standardization in completing the records, thus offering some room for the researcher’s interpretation bias. The academic data could not be directly assessed by the present researchers, but were collected by a professional from the Academic Board. The Academic Board provided data using their own categorization criteria, which were subsequently re-categorized by the researchers.

## CONCLUSION

The students who underwent psychiatric treatment were the most severely affected group among the individuals who sought the campus mental health care service. They represented around 15% of the students who were assisted at the service, and included people diagnosed with severe mental disorders. The academic performance indicators found in this group did not differ radically from those of the control group. In the case of the undergraduates, their course completion rates were even somewhat better, which may suggest that there is a positive effect from care with regard to prevention of course abandonment.
